# Regulatory effects of hawthorn on lipid metabolic homeostasis: mechanisms, evidences, and perspectives

**DOI:** 10.3389/fnut.2026.1807050

**Published:** 2026-03-11

**Authors:** Hefang Xu, Xinyue Zhao, Hui Bai, Shasha Li

**Affiliations:** School of Pharmacy, Qilu Medical University, Zibo, Shandong, China

**Keywords:** chemical constituents, gut microbiota, hawthorn, lipid metabolism, medicinal and edible homologous plants

## Abstract

Dysregulation of lipid metabolic homeostasis is a central pathological feature of metabolic disorders, including obesity and non-alcoholic fatty liver disease. Owing to the limitations of current pharmacological therapies, safe and effective natural interventions are increasingly sought. Hawthorn, a traditional medicinal and edible plant, contains diverse bioactive constituents such as flavonoids, phenylpropanoids, terpenoids, and polysaccharides, and has shown considerable potential in regulating lipid metabolism. Recent studies demonstrate that hawthorn improves lipid metabolic homeostasis through multiple mechanisms, including suppression of hepatic lipogenesis, enhancement of fatty acid *β*-oxidation, improvement of insulin signaling, regulation of adipose tissue function, and modulation of cholesterol and bile acid metabolism. In addition, hawthorn participates in the regulation of lipid metabolism by reshaping gut microbiota composition and influencing gut–liver axis signaling. Evidence from *in vitro* and *in vivo* studies, together with limited clinical investigations, indicates that hawthorn exhibits favorable safety profiles and metabolic regulatory effects, supporting its potential application in functional foods and nutritional interventions. Nevertheless, current research is limited by suboptimal experimental models, incomplete mechanistic integration, and insufficient high-quality clinical evidence. Future studies should incorporate multi-omics approaches and well-designed clinical trials to further elucidate the core targets and causal mechanisms underlying hawthorn-mediated lipid metabolic regulation.

## 1 Introduction

Lipid metabolic homeostasis is a fundamental physiological process essential for maintaining energy balance and metabolic health, encompassing multiple dynamic processes, including lipid uptake, synthesis, catabolism, transport, and storage. Disruption of this homeostasis frequently leads to the onset and progression of a spectrum of metabolism-related disorders, such as dyslipidemia, obesity, non-alcoholic fatty liver disease (NAFLD), insulin resistance, and atherosclerosis (1, 2). Lipid metabolic disorders have thus emerged as a major threat to public health. At present, clinical management of dyslipidemia and related diseases primarily relies on pharmacological agents such as statins, fibrates, and cholesterol absorption inhibitors (3). Although these agents demonstrate well-established efficacy in lowering lipid levels and reducing cardiovascular events, their long-term use is associated with several limitations, including myotoxicity, hepatic dysfunction, poor tolerability, and suboptimal therapeutic responses in certain patient populations (4–6). Consequently, the development of safer strategies suitable for long-term intervention to restore and maintain lipid metabolic homeostasis has become a key focus in contemporary metabolic disease research.

In recent years, bioactive compounds derived from natural plants have attracted increasing attention due to their multitarget regulatory properties, relatively low toxicity, and suitability for long-term use as functional foods or dietary supplements. Plant-derived bioactive substances, including flavonoids, phenylpropanoids and terpenoids, exhibit substantial potential in regulating lipid metabolism, alleviating inflammatory responses, and enhancing metabolic flexibility (7). Against this backdrop, the modern metabolic regulatory functions of traditional medicinal and edible plants have emerged as a major research focus. Hawthorn (*Crataegus* spp.), a traditional food–medicine resource widely used in Asia, has long been employed for digestive regulation and circulation improvement. Contemporary studies further demonstrate its diverse biological activities, including lipid-lowering effects, cardiovascular protection, and antioxidant properties (8). Accumulating evidence indicates that hawthorn and its extracts participate in the fine regulation of lipid metabolic homeostasis through multiple mechanisms, including modulation of lipid synthesis and oxidation, improvement of adipose tissue function, regulation of cholesterol metabolism, and remodeling of gut microbiota composition (9–11). These findings suggest that hawthorn is not merely a traditional lipid-lowering agent but may also serve as a natural intervention with broad potential for systemic metabolic regulation. It is important to note that hawthorn, as a natural intervention with both medicinal and edible properties, has lower toxicity and higher safety compared to chemical lipid-lowering drugs. Long-term consumption does not cause obvious adverse reactions, and it can simultaneously exert cardiovascular protective effects such as antioxidant activity and blood pressure regulation. Additionally, hawthorn is suitable for daily dietary supplementation, with a milder mode of use, making it more appropriate as a daily conditioning method for cardiovascular health.

On this basis, this review systematically surveys studies published over the past 5 years by retrieving literature from PubMed, CNKI, and Web of Science using the keywords “hawthorn” and “lipid metabolism.” The review aims to comprehensively summarize recent advances in the regulation of lipid metabolic homeostasis by hawthorn, with a particular focus on its phytochemical basis, key molecular mechanisms, gut microbiota-mediated effects, and evidence from *in vitro*, *in vivo*, and clinical studies. In addition, the safety profile, application potential, and future research directions of hawthorn are discussed. By integrating and critically evaluating the available evidence, this review seeks to provide a theoretical foundation and a reference framework for the scientific application of hawthorn in the prevention and management of lipid metabolism-related diseases, as well as for future research endeavors.

## 2 Biological basis of lipid metabolic homeostasis

Lipid metabolic homeostasis refers to the ability of the organism to maintain a dynamic balance between lipid supply and demand under varying nutritional and energy states through precise regulation of lipid absorption, synthesis, catabolism, transport, and storage. This process requires coordinated regulation among multiple organs, including the intestine, liver, adipose tissue, and skeletal muscle, and is tightly controlled by integrated neural, hormonal, and metabolic signaling networks (12, 13). Disruption of lipid metabolic homeostasis is widely recognized as a shared pathological basis underlying a range of metabolic diseases.

### 2.1 Lipid absorption, synthesis, catabolism, and transport

Dietary lipids are primarily digested and absorbed in the small intestine, enter the circulation in the form of chylomicrons, and are subsequently distributed to the liver and peripheral tissues. The liver serves as the central hub of lipid metabolism, being responsible for the redistribution of exogenous lipids as well as for governing endogenous lipid synthesis (14). Under conditions of energy surplus, glucose and fructose are converted into fatty acids via acetyl-coenzyme A, which are subsequently esterified into triglycerides for storage or exported in the form of very-low-density lipoproteins (VLDL) (15, 16). Adipose tissue represents the largest energy storage organ in the body and maintains energy buffering capacity through the synthesis and breakdown of triglycerides within adipocytes (17). During fasting or physical activity, triglycerides stored in adipose tissue undergo hydrolysis mediated by hormone-sensitive lipase (HSL) and adipose triglyceride lipase (ATGL), releasing free fatty acids (FFA) for oxidative utilization by peripheral tissues (18, 19). When lipid intake and synthesis chronically exceed the capacity for lipid catabolism and oxidation, ectopic lipid accumulation occurs in non-adipose tissues such as the liver, skeletal muscle, and heart, thereby inducing lipotoxicity and metabolic dysfunction (20).

### 2.2 Central regulatory role of the liver in lipid metabolic homeostasis

The liver plays a pivotal role in lipid metabolic homeostasis by functioning as a metabolic “sensor–integrator–distributor” (21, 22). On the one hand, hepatocytes sense nutritional status and hormonal signals, such as insulin and glucagon, thereby regulating fatty acid synthesis, oxidation, and cholesterol metabolism (23). On the other hand, the liver influences systemic lipid distribution and intestinal lipid absorption through the secretion of lipoproteins and bile acids (24). Under conditions of insulin resistance, hepatic lipid metabolic regulation becomes profoundly disrupted, characterized by persistent activation of fatty acid synthesis, suppression of fatty acid oxidation, and increased VLDL secretion, ultimately leading to hypertriglyceridemia and hepatic steatosis (25). Consequently, hepatic lipid metabolic dysfunction is regarded as a critical pathological link connecting dietary factors to systemic lipid metabolic disorders.

### 2.3 Key transcription factors and signaling pathways regulating lipid metabolic homeostasis

The fine regulation of lipid metabolic homeostasis relies on the coordinated actions of multiple transcription factors and energy-sensing signaling pathways. Among these, AMP-activated protein kinase (AMPK) is regarded as a central sensor of cellular energy status and is activated under conditions of energy deprivation, thereby suppressing fatty acid synthesis, promoting fatty acid oxidation, and improving mitochondrial function (26, 27). Activation of AMPK rapidly reduces lipogenesis by inhibiting acetyl-coenzyme A carboxylase (ACC) and downstream fatty acid synthetic pathways (28). Sterol regulatory element-binding protein 1c (SREBP-1c) is a key transcription factor governing fatty acid and triglyceride synthesis, and its activity is markedly enhanced under conditions of high glucose and hyperinsulinemia (29, 30). Persistent activation of SREBP-1c is considered a major molecular mechanism underlying non-alcoholic fatty liver disease and hyperlipidemia. The peroxisome proliferator-activated receptor (PPAR) family also plays a central role in maintaining lipid metabolic homeostasis. Specifically, PPARα primarily promotes fatty acid *β*-oxidation and energy expenditure, whereas PPARγ plays a dominant role in adipocyte differentiation and lipid storage (31, 32). Dysregulation of PPAR signaling frequently results in abnormal lipid distribution and reduced metabolic flexibility. In addition, nuclear receptors such as the farnesoid X receptor (FXR) and liver X receptor sense bile acid and cholesterol levels to regulate cholesterol transport, lipid synthesis, and inflammatory responses, thereby playing critical roles in lipid metabolic regulation along the gut–liver axis (33).

### 2.4 Integrative role of the gut–liver axis in lipid metabolic homeostasis

**Figure 1:**
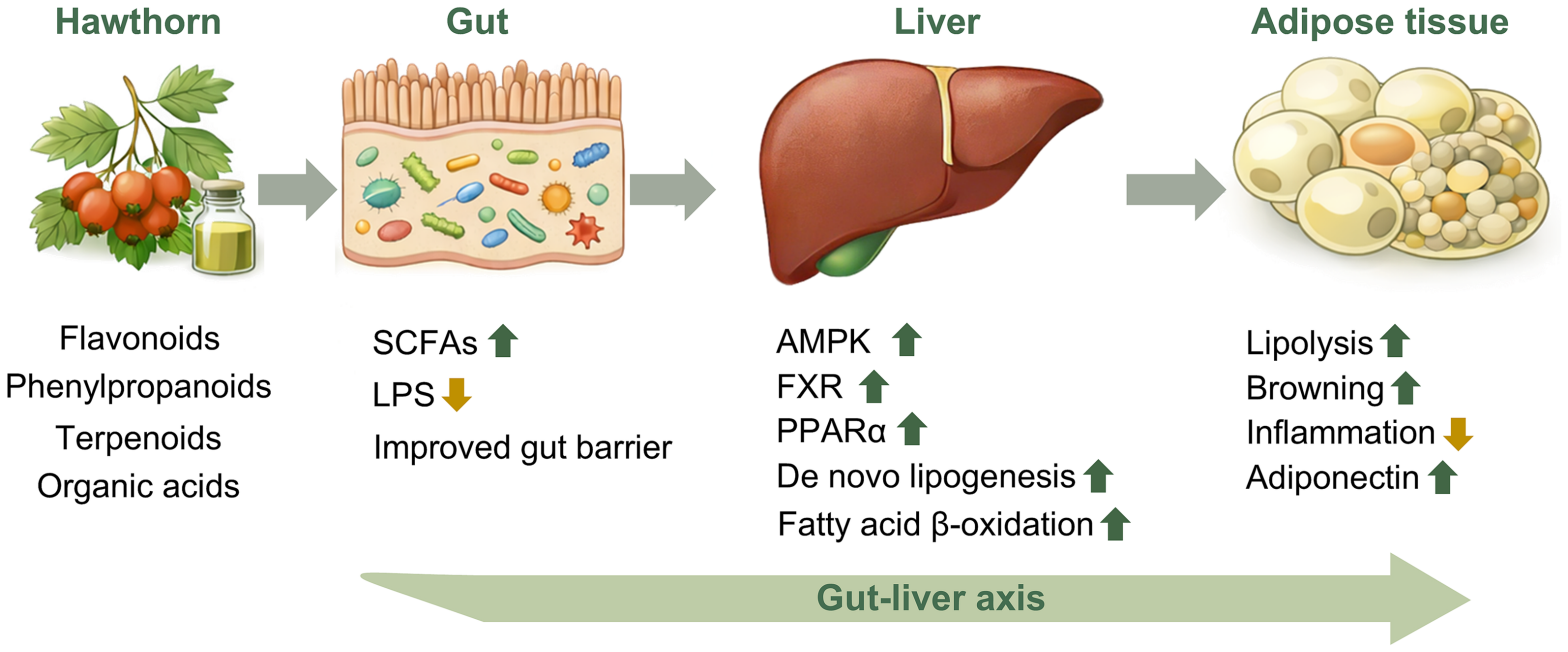
Proposed mechanisms by which hawthorn regulates lipid metabolic homeostasis through the gut–liver–adipose tissue axis (SCFAs, short chain fatty acids; LPS, lipopolysaccharide; AMPK, AMP-activated protein kinase; FXR, farnesoid X receptor; PPARα, peroxisome proliferator-activated receptor alpha).

Recent studies indicate that lipid metabolic homeostasis is not governed by isolated organ-specific regulation but rather emerges from a dynamic network involving the intestine, liver, and adipose tissue, interconnected through metabolites, hormonal signals, and immune mediators (34). The gut microbiota plays an integral role in host lipid metabolism by producing short-chain fatty acids, modulating bile acid profiles, and influencing inflammatory status (35, 36). Dysfunction of the gut–liver axis can result in aberrant bile acid signaling, impaired intestinal barrier integrity, and low-grade chronic inflammation, thereby exacerbating lipid metabolic dysregulation. This conceptual framework provides an important biological basis for understanding how natural plant-derived compounds may improve lipid metabolic homeostasis through modulation of the gut microbiota (Figure 1).

## 3 Phytochemical basis of hawthorn

Hawthorn (*Crataegus* spp.), belonging to the genus Crataegus of the family Rosaceae, is a traditional plant resource with both edible and medicinal value and has been used for centuries in China, Europe, and North America. Hawthorn has been widely applied for improving digestive function, regulating blood lipid levels, and preventing cardiovascular diseases (37). With advances in modern analytical and omics technologies, a wide range of bioactive chemical constituents in hawthorn have been systematically identified, and their potential roles in regulating lipid metabolic homeostasis have attracted increasing attention. Phytochemical studies indicate that hawthorn contains diverse bioactive compounds closely associated with lipid metabolism regulation, primarily including flavonoids, phenylpropanoids, and terpenoids (38).

### 3.1 Flavonoids

Flavonoids represent the most extensively investigated class of bioactive constituents in hawthorn and are characterized by pronounced antioxidant, anti-inflammatory, and metabolic regulatory activities. Recent studies have demonstrated that both hawthorn leaves and fruits are rich sources of flavonoid compounds. In hawthorn fruits, which constitute the primary medicinal and edible part, more than 20 flavonoids have been identified, including rutin and hyperoside (Table 1).

**Table 1:** Flavonoids identified in hawthorn fruits.

No.	Chemical name	Molecular formula	Reference
1	Quercetin	C_15_H_10_O_7_	(80)
2	Hyperoside	C_21_H_20_O_12_	(81)
3	Isoquercitrin	C_21_H_20_O_12_	(81)
4	Rutin	C_27_H_30_O_16_	(82)
5	Pinnatifidin	C_15_H_18_O_3_	(83)
6	Vitexin	C_21_H_20_O_10_	(84)
7	Vitexin-2″-O-rhamnoside	C_27_H_30_O_14_	(85)
8	Naringin-O-hexoside	C_27_H_32_O_14_	(80)
9	Phloretin-C-hexoside	C_27_H_34_O_15_	(80)
10	Cyanidin-3-galactoside	C_21_H_21_ClO_11_	(86)
11	Ideain	C_21_H_21_ClO_11_	(87)
12	Epicatechin	C_15_H_14_O_6_	(88)
13	Procyanidin B5	C_30_H_26_O_12_	(89)
14	Procyanidin B2	C_30_H_26_O_12_	(88)
15	Procyanidin B4	C_30_H_26_O_12_	(90)
16	Procyanidin D1	C_30_H_26_O_13_	(90)
17	Procyanidin C1	C_45_H_38_O_18_	(89)
18	Procyanidin trimer I	C_45_H_38_O_18_	(91)
19	Procyanidin trimer II	C_45_H_38_O_18_	(91)
20	Procyanidin trimer III	C_45_H_38_O_18_	(91)
21	Procyanidin tetramer I	C_60_H_50_O_24_	(89)
22	Procyanidin tetramer II	C_60_H_50_O_24_	(89)
23	Procyanidin pentamer I	C_75_H_62_O_30_	(89)
24	Procyanidin pentamer II	C_75_H_62_O_30_	(89)
25	Procyanidin dimerhexoside	C_36_H_36_O_17_	(86)
26	Procyanidin dimer I	C_30_H_26_O_12_	(86)
27	Hexamer F	C_90_H_74_O_36_	(90)

### 3.2 Phenylpropanoids

Phenylpropanoids represent another important class of constituents in hawthorn fruits. Compounds belonging to this group exhibit diverse biological activities, including antioxidant, anti-inflammatory, and antitumor effects. To date, more than 90 phenylpropanoid compounds have been identified in hawthorn fruits (Table 2).

**Table 2:** Phenylpropanoids identified in hawthorn fruits.

No.	Chemical name	Molecular formula	Reference
1	Chlorogenic acid	C_16_H_18_O_9_	(92)
2	Caffeic acid	C_9_H_8_O_4_	(80)
3	Isochlorogenic acid A	C_25_H_24_O_12_	(80)
4	Caffeoylshikimic acid	C_16_H_18_O_9_	(80)
5	(−)-Crataegusoid A	C_29_H_32_O_10_	(93)
6	(+)-Crataegusoid A	C_29_H_32_O_10_	(93)
7	Crataegusoid B	C_21_H_24_O_7_	(93)
8	Crataegusoid C	C_20_H_22_O_6_	(93)
9	Crataegusoid D	C_22_H_26_O_8_	(93)
10	(−)-Crataegusoid E	C_28_H_30_O_9_	(93)
11	(+)-Crataegusoid E	C_28_H_30_O_9_	(93)
12	Crataegusoid F	C_19_H_20_O_5_	(93)
13	Dihydrosinapyl alcohol	C_11_H_16_O_4_	(94)
14	Dihydroconiferyl alcohol	C_10_H_14_O_3_	(94)
15	3-Hydroxy-1-(4-hydroxy-3,5-dimethoxyphenyl) propan-1-one	C_11_H_14_O_5_	(94)
16	2-[4-(3-Ydroxypropyl)-2-methoxyphenoxy]-1,3propanediol	C_12_H_18_O_6_	(94)
17	3-(3,4,5-Trimethoxyphenyl) propan-1-ol	C_12_H_18_O_4_	(94)
18	ω-Hydroxypropioguaiacone	C_10_H_12_O_4_	(94)
19	Propiosyringone	C_11_H_14_O_4_	(94)
20	Crataegusins A	C_18_H_14_O_8_	(95)
21	Crataegusins B	C_19_H_14_O_9_	(95)
22	Cratapinnatifidas A	C_21_H_26_O_9_	(96)
23	Cratapinnatifidas B	C_21_H_26_O_9_	(96)
24	Cratapinnatifidas C	C_21_H_26_O_9_	(96)
25	Cratatifida A	C_21_H_26_O_9_	(97)
26	Cratatifida B	C_20_H_24_O_8_	(97)
27	Cratatifida C	C_20_H_24_O_8_	(97)
28	Cratatifida D	C_20_H_24_O_8_	(97)
29	Cratatifida E	C_20_H_24_O_8_	(97)
30	Cratatifida F	C_20_H_24_O_8_	(97)
31	Cratatifida G	C_20_H_24_O_8_	(97)
32	Cratatifida H	C_20_H_24_O_8_	(97)
33	Cratatifida I	C_20_H_24_O_8_	(97)
34	Cratatifida J	C_20_H_24_O_8_	(97)
35	Crasesquineolignan A	C_40_H_46_O_10_	(98)
36	Crasesquineolignan B	C_30_H_34_O_8_	(98)
37	Crasesquineolignan C	C_22_H_26_O_7_	(98)
38	Crasesquineolignan D	C_21_H_24_O_6_	(98)
39	Crasesquineolignan E	C_19_H_22_O_5_	(98)
40	Crasesquineolignan F	C_20_H_24_O_5_	(98)
41	Crasesquineolignan G	C_21_H_26_O_5_	(98)
42	(+)-Crataegusanoid A	C_25_H_30_O_7_	(94)
43	(−)-Crataegusanoid A	C_25_H_30_O_7_	(94)
44	(+)-Crataegusanoid B	C_24_H_28_O_7_	(94)
45	(−)-Crataegusanoid B	C_24_H_28_O_7_	(94)
46	(+)-Crataegusanoid C	C_26_H_32_O_8_	(94)
47	(−)-Crataegusanoid C	C_26_H_32_O_8_	(94)
48	(+)-Crataegusanoid D	C_23_H_26_O_7_	(94)
49	(−)-Crataegusanoid D	C_23_H_26_O_7_	(94)
50	Crataeguslignan A	C_38_H_44_O_9_	(99)
51	4”-O-(8-guaiacylglycerol) buddlenol A	C_47_H_52_O_11_	(99)
52	Crataegusanoid E	C_22_H_24_O_6_	(94)
53	Crataegusanoid F	C_21_H_22_O_5_	(94)
54	Lariciresinol	C_20_H_24_O_6_	(94)
55	5′-Methoxylariciresinol	C_21_H_26_O_6_	(94)
56	Epipinoresinol	C_20_H_22_O_6_	(94)
57	Pinoresinol	C_20_H_22_O_6_	(94)
58	Medioresinol	C_21_H_24_O_7_	(94)
59	Syringarenol	C_19_H_20_O_4_	(94)
60	Lyoniresinol	C_21_H_26_O_8_	(100)
61	(+)-Crataegusin A	C_40_H_44_O_12_	(101)
62	(−)-Crataegusin A	C_40_H_44_O_12_	(101)
63	(+)-(7S,8R)-crataegusin B	C_38_H_42_O_11_	(101)
64	(−)-(7R,8S)-crataegusin B	C_38_H_42_O_11_	(101)
65	(+)-(7S,8R)-crataegusin C	C_37_H_40_O_11_	(101)
66	(−)-(7R,8S)-crataegusin C	C_37_H_40_O_11_	(101)
67	(+)-7S,8R-crataegifin A	C_39_H_44_O_12_	(102)
68	(−)-7R,8S-crataegifin A	C_39_H_44_O_12_	(102)
69	(+)-Crataegifin B	C_38_H_42_O_12_	(102)
70	(−)-Crataegifin B	C_38_H_42_O_12_	(102)
71	(+)-7S,8R-crataegifin C	C_37_H_40_O_12_	(102)
72	(−)-7R,8S-crataegifin C	C_37_H_40_O_12_	(102)
73	(+)-7S,8R-crataegifinD	C_36_H_38_O_12_	(102)
74	(−)-7R,8S-crataegifin D	C_36_H_38_O_12_	(102)
75	(+)-Crataegusal A	C_21_H_20_O_10_	(103)
76	(−)-Crataegusal A	C_21_H_20_O_10_	(103)
77	(+)-Crataegusal B	C_20_H_18_O_9_	(103)
78	(−)-Crataegusal B	C_20_H_18_O_9_	(103)
79	(+)-7S,8S-4,7,9,9′-tetrahydroxy-3,3′,5′-trimethoxy-8-O-4′-neolignan	C_22_H_28_O_10_	(104)
80	(−)-7R,8R-4,7,9,9′-tetrahydroxy-3,3′,5′-trimethoxy-8-O-4′-neolignan	C_22_H_28_O_10_	(104)
81	(+)-7S,8R-4,7,9,9′-tetrahydroxy-3,3′,5′-trimethoxy-8-O-4′-neolignan	C_22_H_28_O_10_	(104)
82	(−)-7R,8S-4,7,9,9′-tetrahydroxy-3,3′,5′-trimethoxy-8-O-4′-neolignan	C_22_H_28_O_10_	(104)
83	(+)-7R,8S-4,7,9,9′-tetrahydroxy-3,5,3′,5′- tetramethoxy-8-O-4′-neolignan	C_23_H_30_O_10_	(104)
84	(−)-7S,8R-4,7,9,9′-tetrahydroxy-3,5,3′,5′- tetramethoxy-8-O-4′-neolignan	C_23_H_30_O_10_	(104)
85	(+)-7S,8S-guaiacylglycerol-8-acetovanillone ether	C_23_H_28_O_8_	(104)
86	(−)-7R,8R-guaiacylglycerol-8-acetovanillone ether	C_23_H_28_O_8_	(104)
87	(+)-7S,8S-guaiacylglycerol 8-vanillin ether	C_21_H_24_O_7_	(104)
88	(−)-7R,8R-guaiacylglycerol 8-vanillin ether	C_21_H_24_O_7_	(104)
89	Evofolin B	C_20_H_18_O_10_	(105)
90	Crataequinone A	C_15_H_10_O_6_	(106)

### 3.3 Terpenoids, organic acids, and other constituents

Hawthorn is rich in a variety of constituents, including terpenoids and organic acids. Terpenoid compounds identified in various plant species have been shown to exert lipid-regulatory and anti-inflammatory effects, as well as to improve insulin sensitivity. Although organic acids, together with pectin, dietary fiber, and small amounts of phytosterols, exhibit relatively modest effects when acting individually, they may synergistically contribute to the regulation of lipid metabolic homeostasis by improving the intestinal environment and inhibiting cholesterol absorption (Table 3).

**Table 3:** Terpenoids, organic acids, and other constituents identified in hawthorn fruits.

No.	Chemical name	Molecular formula	Reference
1	Corosolic acid	C_30_H_48_O_4_	(107)
2	Maslinic acid	C_30_H_48_O_4_	(108)
3	Oleanolic acid	C_30_H_48_O_3_	(109)
4	Ursolic acid	C_30_H_48_O_3_	(107)
5	Euscapic acid	C_29_H_46_O_5_	(107)
6	Linolenic acid	C_18_H_30_O_2_	(100)
7	Lauric acid	C_12_H_24_O_2_	(100)
8	Ascorbic acid	C_6_H_8_O_6_	(100)
9	Caffeic acid	C_9_H_8_O_4_	(100)
10	Benzoic acid	C_7_H_6_O_2_	(100)
11	Citric acid	C_6_H_8_O_7_	(80)
12	Methylcitrate	C_7_H_10_O_7_	(80)
13	Aconitic acid	C_6_H_6_O_6_	(80)
14	Royal jelly acid	C_10_H_18_O_3_	(80)
15	Vanillic acid-C-hexoside	C_13_H_16_O_8_	(80)
16	Malic acid	C_4_H_6_O_5_	(110)
17	Quinic acid	C_7_H_12_O_6_	(110)
18	Epicatechin gallate	C_22_H_18_O_10_	(110)
19	Catechin-C-hexoside	C_21_H_24_O_10_	(110)
20	Mannitol	C_6_H_14_O_6_	(80)
21	Glucose	C_6_H_12_O_6_	(110)
22	Sucrose	C_12_H_22_O_11_	(110)
23	Sorbitol	C_6_H_14_O_6_	(110)
24	Myo-inositol	C_6_H_12_O_6_	(110)
25	Fructose	C_6_H_12_O_6_	(110)
26	Shanyenoside A	C_48_H_76_O_19_	(80)
27	Protocatechuic aldehyde	C_7_H_6_O_3_	(111)
28	Amygdalin	C_20_H_27_NO_11_	(112)

## 4 Mechanisms underlying the effects of hawthorn on lipid metabolic homeostasis

Accumulating evidence indicates that hawthorn does not exert lipid-lowering effects via a single target; rather, it participates systemically in the maintenance of lipid metabolic homeostasis through coordinated regulation across multiple signaling pathways and organ systems. These regulatory effects are primarily manifested through the suppression of lipid synthesis, enhancement of fatty acid oxidation, modulation of adipose tissue function, and improvement of cholesterol and bile acid metabolism.

### 4.1 Inhibition of hepatic lipid synthesis

In the early stages of lipid metabolic homeostasis disruption, aberrant activation of hepatic lipid synthetic pathways often precedes overt lipid accumulation and histopathological alterations. Particularly under conditions of high-sugar and high-fat diet (HFD) or insulin resistance, persistent enhancement of *de novo* lipogenesis is regarded as one of the central driving forces underlying the initiation and progression of non-alcoholic fatty liver disease (39). AMPK, a key sensor of cellular energy status, plays a pivotal role in suppressing hepatic lipid synthesis. AMPK activation not only reflects changes in the intracellular ATP/AMP ratio but also integrates nutritional, hormonal, and stress-related signals to coordinate overall energy metabolic fluxes (40). At the molecular level, activated AMPK directly phosphorylates ACC, thereby inhibiting its catalytic conversion of acetyl-coenzyme A to malonyl-coenzyme A. Because malonyl-coenzyme A serves as a critical substrate for fatty acid synthesis and simultaneously acts as an endogenous inhibitor of carnitine palmitoyltransferase 1 (CPT1), suppression of ACC activity induces metabolic remodeling at both the “synthetic” and “oxidative” ends of lipid metabolism (41, 42).

The study has shown that leucocyanidin, a flavanol compound derived from hawthorn, markedly ameliorates metabolic dysfunction-associated steatotic liver disease. In HFD mice and FFA-induced hepatocyte models, leucocyanidin reduces hepatic lipid accumulation, inflammation, and oxidative stress, while restoring mitochondrial function. Mechanistically, leucocyanidin directly activates the AMPK signaling pathway, enhancing the phosphorylation of AMPK and its downstream target ACC. This activation suppresses the expression of the fatty acid uptake protein CD36 and lipogenic enzymes such as fatty acid synthase (FAS) and diacylglycerol acyltransferase 2, while relieving inhibition of CPT1. Consequently, fatty acid transport into mitochondria for *β*-oxidation is promoted, leading to a reduction in hepatic lipid accumulation (43). In addition, leucocyanidin activates the nuclear factor erythroid 2-related factor 2 (Nrf2)-mediated antioxidant pathway by promoting Nrf2 nuclear translocation and upregulating downstream antioxidant genes, including NAD(P)H quinone dehydrogenase 1 and heme oxygenase-1. This response markedly reduces reactive oxygen species production and lipid peroxidation, alleviates oxidative stress-induced mitochondrial damage, and restores mitochondrial membrane potential and structural integrity (43). Using the HFD-induced mouse model, the effects and underlying mechanisms of hawthorn-derived pectic oligosaccharides (POS) on lipid metabolism were investigated. The results demonstrated that POS significantly reduced serum triglycerides, FFA, total cholesterol, and low-density lipoprotein cholesterol levels. POS also suppressed the expression of inflammatory cytokines tumor necrosis factor-alpha (TNF-α) and interleukin-6 (IL-6) in white adipose tissue and downregulated the gene expression of the macrophage marker CD68, thereby alleviating HFD-induced adipose tissue inflammation. Moreover, POS markedly downregulated the gene expression of key fatty acid synthesis enzymes (ACC and FAS) and triglyceride synthesis-related enzymes [stearoyl-CoA desaturase-1 (SCD-1) and diacylglycerol O-acyltransferase 1 (DGAT1)], while upregulating genes associated with fatty acid oxidation [Acyl-CoA oxidase (ACO) and CPT1 and triglyceride hydrolysis (ATGL and HSL)], thereby suppressing lipogenesis and promoting lipid breakdown and oxidation. These metabolic benefits were associated with activation of the adiponectin-mediated AdipoR1/AMPK/PPARα signaling pathway (44).

In addition, AMPK suppresses the transcriptional expression and maturation of SREBP-1c (45). SREBP-1c is a central transcription factor that drives the expression of lipogenic genes such as FAS and SCD1, and its aberrant activation is closely associated with the extent of hepatic lipid accumulation (46, 47). The animal study has demonstrated that hawthorn total flavonoids (HTF) exert pronounced lipid-lowering effects in HFD-induced hyperlipidemic mice and in FFA-induced HepG2 cell models. Specifically, HTF significantly reduces triglyceride (TG), total cholesterol (TC), and low-density lipoprotein cholesterol (LDL-C) levels in serum and liver, attenuates body weight gain and hepatic lipid accumulation, improves liver function indices [aspartate aminotransferase (AST) and lactate dehydrogenase], alleviates histopathological liver damage, and enhances systemic antioxidant capacity (48). Mechanistically, HTF suppresses lipid and cholesterol synthesis by activating the AMPK/SREBP-1c signaling pathway. In parallel, HTF acts as a natural agonist of PPARα, thereby activating the PPARα/PGC-1α/CPT1A axis to enhance mitochondrial fatty acid *β*-oxidation and energy metabolism. Moreover, HTF reshapes gut microbiota composition by reducing the Firmicutes-to-Bacteroidetes ratio, upregulating CYP7A1 expression, and promoting bile acid circulation, thereby synergistically ameliorating gut–liver axis dysfunction (48).

### 4.2 Improvement of dysregulated coupling between insulin signaling and lipid metabolism

In the context of metabolic diseases, disruption of lipid metabolic homeostasis is frequently accompanied by pronounced insulin resistance. Notably, insulin resistance does not uniformly suppress all downstream insulin signaling pathways but instead manifests as so-called “selective insulin resistance,” characterized by impaired glucose uptake and suppression of gluconeogenesis, while lipogenic signaling remains persistently active (49, 50). In this process, the insulin signaling axis is considered a critical molecular bridge linking insulin resistance to dysregulated lipid metabolism (51).

A study has demonstrated that continuous supplementation with hawthorn proanthocyanidins (HPC) for 8 weeks significantly reduces lipid accumulation in both serum and liver in the HFD-induced lipid metabolic disorder (LMD) rat model, alleviates insulin resistance and oxidative stress, and preserves hepatic tissue architecture. Mechanistically, HPC suppresses HFD-induced elevations in endotoxin (lipopolysaccharide, LPS) levels, modulates the hepatic NF-κB pathway to attenuate inflammatory responses, and activates AMPK signaling to inhibit the expression of lipogenesis-related proteins such as SREBP-1c, thereby reducing hepatic lipid accumulation (52). Gu et al. (53) reported that whole-body exposure to fine particulate matter (PM_2.5_) for 8 weeks disrupts glucose homeostasis and reduces insulin sensitivity in mice, resulting in elevated serum TG levels but decreased hepatic TG content, accompanied by suppressed hepatic fatty acid uptake and lipogenesis and enhanced lipid catabolism and export. Intervention with hawthorn flavonoids (HF) counteracted these alterations by upregulating hepatic fatty acid–binding protein 1 to enhance fatty acid uptake, while downregulating microsomal triglyceride transfer protein and apolipoprotein B expression to reduce lipid export, thereby restoring hepatic TG homeostasis. In addition, HF markedly improved PM_2.5_-induced glucose intolerance and hypertriglyceridemia, leading to a significant attenuation of systemic insulin resistance. Liu et al. (54) identified proanthocyanidin B2, epicatechin, and chlorogenic acid as the major bioactive constituents of hawthorn polyphenol extract (HPE). Continuous administration of HPE at 300 mg/kg for 4 weeks significantly improved body weight, blood glucose, and lipid parameters (TC and TG) in rats with type 2 diabetes, reduced serum insulin and LPS levels, and alleviated pathological damage in skeletal muscle, liver, and aortic tissues. Mechanistically, HPE enhanced insulin signaling by increasing the phosphorylation levels of insulin receptor substrate 1 (IRS1), phosphoinositide 3-kinase (PI3K), protein kinase B (AKT), and glucose transporter type 4 (GLUT4) in the liver, thereby activating the IRS1–PI3K–AKT–GLUT4 pathway and improving insulin resistance.

### 4.3 Promotion of fatty acid oxidation and mitochondrial function

Impaired fatty acid oxidation represents another hallmark of disrupted lipid metabolic homeostasis. In particular, under conditions such as NAFLD and obesity, mitochondrial dysfunction and insufficient oxidative capacity further exacerbate lipid accumulation and lipotoxicity (55–57).

**Figure 2:**
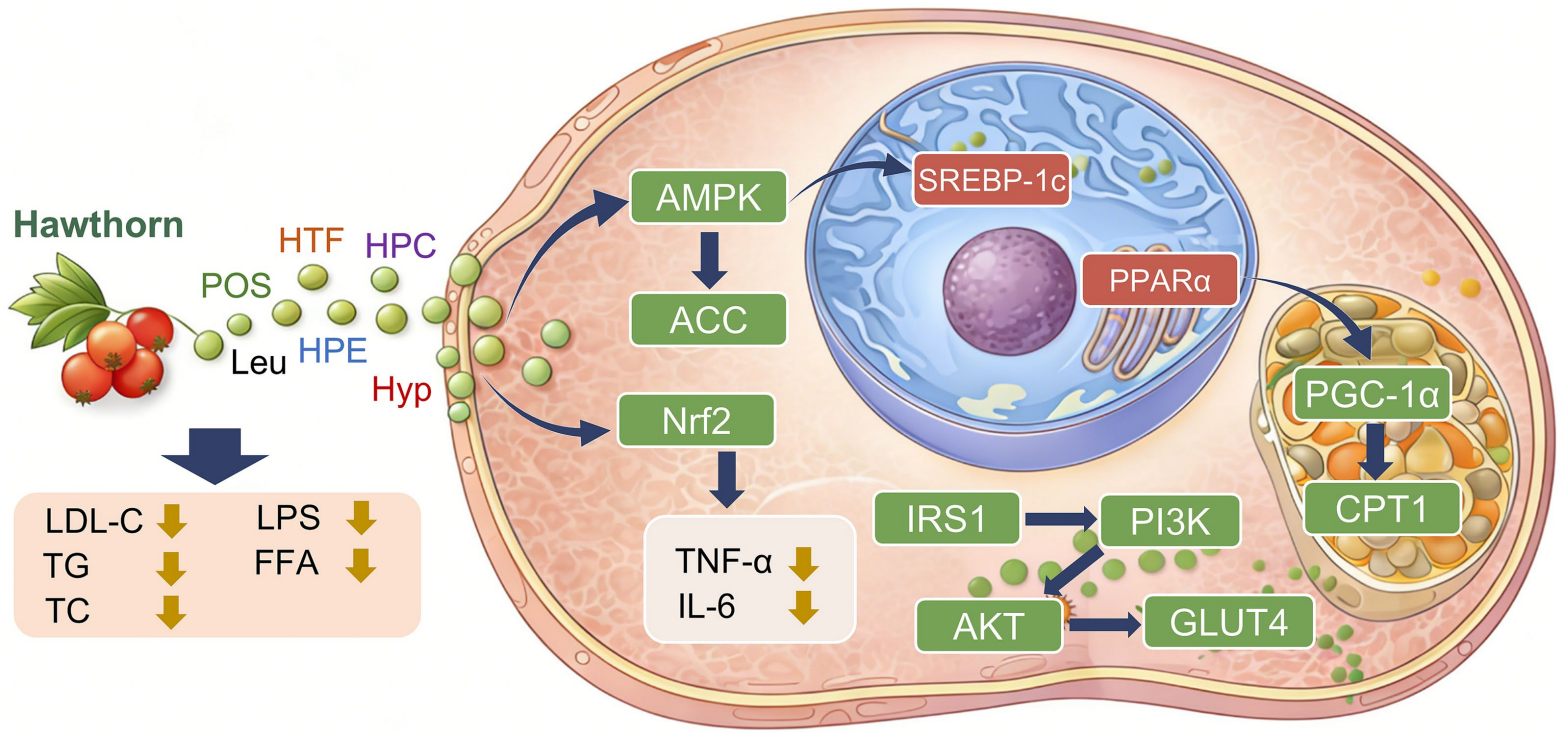
Molecular mechanisms underlying the regulatory effects of hawthorn on lipid metabolic homeostasis (AMPK, AMP-activated protein kinase; ACC, acetyl-coenzyme A carboxylase; Nrf2, nuclear factor erythroid 2-related factor 2; TNF-α, tumor necrosis factor-alpha; IL-6, interleukin-6; LDL-C, low-density lipoprotein cholesterol; LPS, lipopolysaccharide; TC, total cholesterol; TG, triglyceride; FFA, free fatty acids; IRS1, insulin receptor substrate 1; PI3K, phosphoinositide 3-kinase; AKT, protein kinase B; GLUT4, glucose transporter type 4; SREBP-1c, sterol regulatory element-binding protein 1c; PPARα, peroxisome proliferator-activated receptor alpha; CPT1, carnitine palmitoyltransferase 1; PGC-1α, peroxisome proliferator-activated receptor *γ* coactivator 1α; HTF, hawthorn total flavonoids; HPC, hawthorn proanthocyanidins; POS, hawthorn-derived pectic oligosaccharides; HPE, hawthorn polyphenol extract; Leu, leucocyanidin; Hyp, hyperoside).

The study has shown that in HFD-induced rat models, hyperoside directly activates hepatic FXR, thereby promoting bile acid efflux and enhancing fatty acid *β*-oxidation, while concurrently suppressing *de novo* lipogenesis. Mechanistically, hyperoside inhibits fatty acid and triglyceride synthesis via the FXR–SCD1/SREBP1 axis, while independently suppressing ATP-citrate lyase (ACLY), thereby reducing acetyl-coenzyme A availability at its source and synergistically attenuating lipid accumulation (58). Experimental evidence indicates that hawthorn polysaccharides (HP) dose-dependently reduce hepatic lipid deposition in HFD mice, ameliorate dyslipidemia, and attenuate liver injury. Transcriptomic analyses suggest that HP exerts its effects primarily by regulating pathways related to cholesterol and fatty acid metabolism, including sterol biosynthesis, PPAR signaling, and AMPK signaling, as well as immune-associated genes. Metabolomic profiling further demonstrates that HP corrects hepatic metabolic disturbances by modulating pathways such as tyrosine metabolism, inositol phosphate metabolism, and ATP-binding cassette transporter pathways (59). Hawthorn ethanol extract (HEE) markedly reduces hepatic lipid accumulation, improves dyslipidemia, liver dysfunction, and inflammatory status, while promoting triglyceride hydrolysis and suppressing fatty acid synthesis. Mechanistically, HEE reshapes hepatic lipid metabolic profiles by upregulating lipolysis-related genes, including ATGL, HSL, and MGL, while suppressing FAS expression (60). Leucocyanidin exerts dual actions by directly binding allosterically to and activating AMPK, thereby suppressing fatty acid uptake and lipogenesis while promoting fatty acid *β*-oxidation. Concurrently, it activates the Nrf2 antioxidant pathway to alleviate oxidative stress and restore mitochondrial structure and function (43) (Figure 2).

### 4.4 Regulation of adipose tissue differentiation, lipolysis, and the inflammatory microenvironment

Aberrant adipocyte differentiation, dysregulated lipolytic capacity, and chronic low-grade inflammation are considered key pathological bases underlying obesity-associated lipid metabolic imbalance (61).

PPARγ and CCAAT/enhancer-binding protein *α* (C/EBPα) are key transcription factors driving adipocyte differentiation and lipid storage, and their excessive activation promotes abnormal expansion of adipose tissue (62). The study has shown that hawthorn POS significantly reduce body weight gain and serum levels of TG, TC, FFA, and LDL-C, while suppressing the expression of inflammatory cytokines TNF-α and IL-6 and the macrophage marker CD68 in white adipose tissue. Mechanistically, POS downregulates genes involved in fatty acid and triglyceride synthesis, including ACC, FAS, SCD-1, and DGAT1, while upregulating lipolysis- and fatty acid oxidation-related genes such as ATGL, HSL, CPT1, and ACO, thereby suppressing lipogenesis and promoting lipid breakdown. Further the study revealed that POS markedly increases adiponectin levels and activates the AdipoR1/AMPK/PPARα signaling pathway, which represents a key mechanism underlying its lipid metabolic benefits (44). Both *in vivo* and *in vitro* experiments demonstrate that hyperoside significantly reduces body weight and fat mass in HFD-induced obese male C57BL/6 J mice, while improving lipid profiles and glucose tolerance. These effects are primarily attributed to the browning of white adipose tissue and enhanced energy expenditure. Mechanistically, hyperoside inhibits cyclin-dependent kinase 6, thereby promoting dephosphorylation and nuclear translocation of transcription factor EB, which in turn upregulates PGC-1α and uncoupling protein 1 to activate thermogenic programs in adipose tissue. Concurrently, hyperoside robustly induces autophagy–lysosome biogenesis, enhances the colocalization of lipid droplets with LC3 and LAMP1, and promotes lipophagy and ATGL-mediated lipolysis (63). Hawthorn-derived POS significantly reduce hepatic triglyceride and cholesterol deposition in HFD-induced male mice, ameliorate hepatic steatosis, and increase circulating adiponectin (ADPN) levels. In white adipose tissue, POS simultaneously activates the PKA/ERK/CREB and C/EBPα/PPARγ pathways, thereby promoting ADPN synthesis and secretion. The released ADPN subsequently acts on the liver to activate the AdipoR1/AMPKα/PGC-1α and AdipoR2/PPARα signaling pathways, suppressing fatty acid synthesis and enhancing fatty acid *β*-oxidation. Meanwhile, POS upregulates nuclear respiratory factor 1 and mitochondrial transcription factor A, improves mitochondrial function, and further promotes lipid oxidation (64). Hawthorn carbon dots (HCD), synthesized using a green hydrothermal method, exhibit pronounced antioxidant and anti-inflammatory activities, scavenging multiple reactive oxygen species and suppressing the expression of inflammatory cytokines such as TNF-α, IL-6, and IL-1β in RAW264.7 macrophages. *In vivo* experiments demonstrate that HCD markedly attenuates body weight gain, hepatic lipid accumulation, and adipocyte hypertrophy in HFD mice, while improving glucose tolerance and insulin resistance. By alleviating oxidative stress and chronic inflammation, HCD mitigates obesity-associated metabolic damage (65).

### 4.5 Regulation of cholesterol metabolism and bile acid signaling pathways

Cholesterol homeostasis is a critical component of lipid metabolic homeostasis. Its dysregulation not only alters circulating lipid profiles but also influences energy metabolism and inflammatory responses through bile acid-mediated signaling. Hawthorn has long been regarded in traditional medicine as having lipid-regulating and digestive-promoting effects, and modern studies are gradually elucidating its potential molecular basis in cholesterol metabolism (66).

The study has shown that hyperoside, a flavonoid glycoside present in hawthorn, directly activates the FXR, thereby promoting bile acid efflux from the liver to the intestine and enhancing fatty acid oxidation. In parallel, hyperoside directly inhibits ACLY, reducing acetyl-coenzyme A availability and consequently suppressing hepatic *de novo* fatty acid and triglyceride synthesis. At the intestinal level, hyperoside decreases the abundance of bile salt hydrolase (BSH)-producing bacteria, thereby reducing bile acid deconjugation, increasing the proportion of conjugated bile acids, and attenuating bile acid-induced hepatotoxicity. These coordinated effects—FXR activation and ACLY inhibition—synergistically ameliorate hepatic lipid accumulation and bile acid metabolic dysregulation (58). Hawthorn extract (HE) inhibits pancreatic lipase activity through a reversible mixed-type inhibition mechanism and induces conformational changes in the enzyme’s secondary and tertiary structures via non-covalent interactions, thereby impairing its catalytic function. Binding of HE to pancreatic lipase reduces the structural stability of the enzyme. In addition, HE significantly increases cholesterol micelle size and decreases micellar solubility, thereby inhibiting intestinal cholesterol absorption (67). Hawthorn pectin markedly reduces serum total cholesterol and triglyceride levels in HFD-induced hyperlipidemic rats and alleviates liver function impairment. Mechanistic analyses indicate that hawthorn pectin exerts its lipid-lowering effects primarily by promoting fecal excretion of cholesterol and bile acids. Moreover, hawthorn pectin is efficiently fermented by gut microbiota, leading to a marked increase in short-chain fatty acid levels—particularly butyrate—thereby improving the lipid metabolic microenvironment (68).

### 4.6 Regulation of lipid metabolic homeostasis by hawthorn via the gut microbiota

The gut microbiota plays an indispensable regulatory role in maintaining host lipid metabolic homeostasis. Intestinal microorganisms not only participate in the metabolic transformation of dietary lipids and bile acids but also influence hepatic function, adipose tissue activity, and systemic energy metabolism through their metabolites (69). Dysbiosis of the gut microbiota is widely recognized as an important pathological basis underlying metabolic diseases such as obesity, dyslipidemia, and non-alcoholic fatty liver disease.

**Figure 3:**
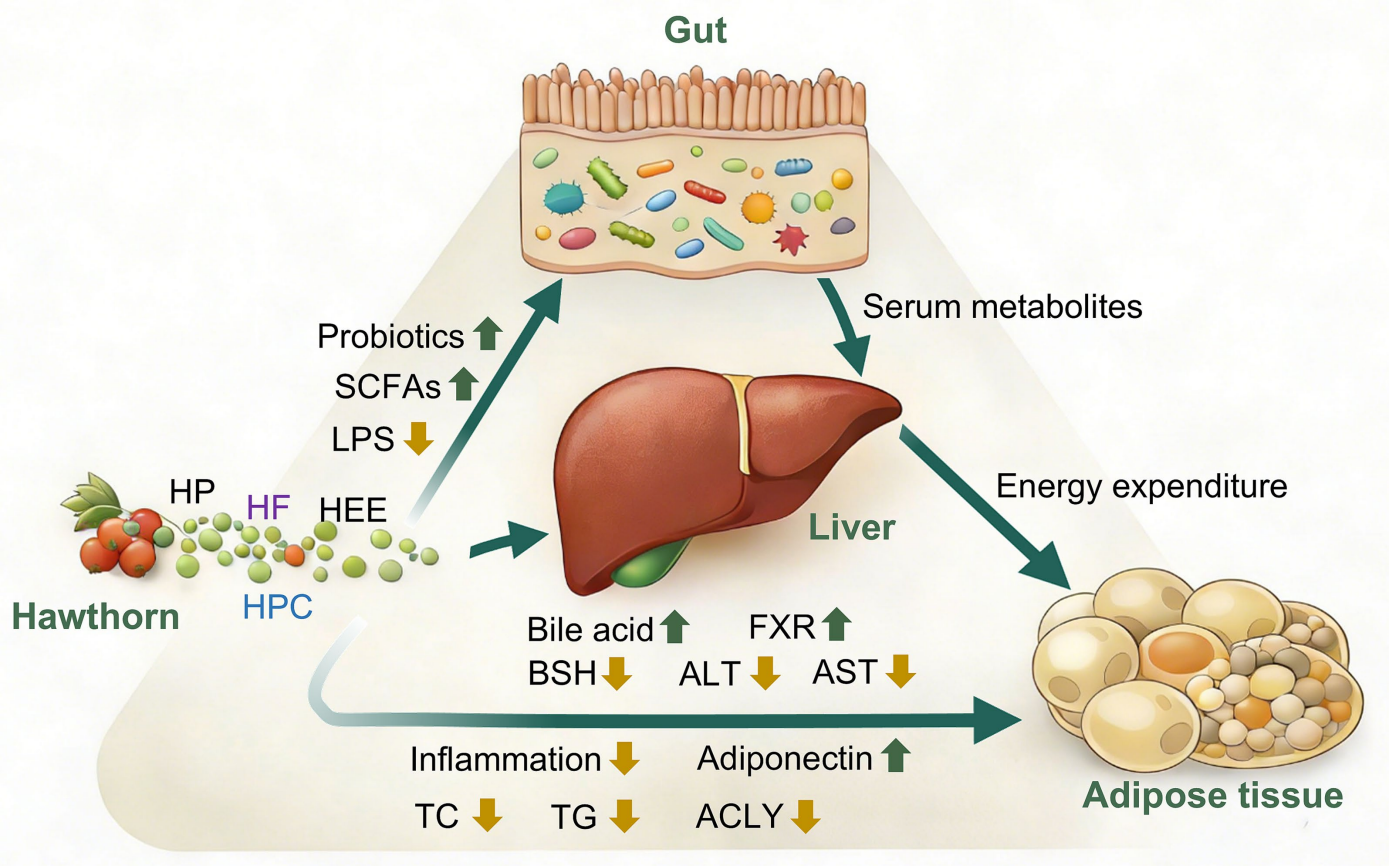
Gut microbiota-involved mechanisms of hawthorn in the regulation of lipid metabolic homeostasis (SCFAs, short chain fatty acids; LPS, lipopolysaccharide; TC, total cholesterol; TG, triglyceride; ACLY, ATP-citrate lyase; ALT, alanine aminotransferase; AST, aspartate aminotransferase; FXR, farnesoid X receptor; BSH, bile salt hydrolase; HP, hawthorn polysaccharides; HF, hawthorn flavonoids; HEE, hawthorn ethanol extract; HPC, hawthorn proanthocyanidins).

HP exhibit pronounced protective effects against HFD-induced NAFLD in mice. A study has shown that HP effectively attenuates body weight gain and hepatic lipid accumulation, improves liver function, and significantly reduces serum alanine aminotransferase (ALT) and AST levels. HP also exerts anti-NAFLD effects by ameliorating hepatic metabolic disturbances and suppressing oxidative stress and inflammatory responses. HP modulates key hepatic metabolites, including amino acids, lipids, and vitamins, thereby partially restoring metabolic abnormalities. In addition, HP alleviates gut microbiota dysbiosis and increases intestinal short-chain fatty acid levels, further supporting its efficacy in ameliorating NAFLD through microbiota-mediated mechanisms (70). Supplementation with HPC has been shown to significantly reduce serum lipid levels in HFD-induced LMD rats, improve insulin resistance, attenuate oxidative stress, and repair hepatic injury. Concurrently, HPC modulates gut microbiota composition, restores intestinal barrier integrity, and reduces the absorption of gut-derived endotoxin LPS, thereby alleviating intestinal and hepatic inflammation (52). Hawthorn flavonoids markedly attenuate body weight gain and lipid accumulation in HFD-induced obese rats. Following flavonoid treatment, gut microbiota composition is significantly improved, particularly with an increased abundance of beneficial taxa such as *Lachnospiraceae*. The genus *Lachnospiraceae* can promote fat oxidation by producing SCFA. Moreover, hawthorn flavonoid intervention effectively modulates serum pathways related to lipid metabolism, bile acid synthesis, and amino acid metabolism, thereby ameliorating metabolic disturbances induced by HFD. Correlation analyses further reveal a close association between alterations in gut microbiota composition and changes in serum metabolites, suggesting that hawthorn flavonoids exert anti-obesity effects by modulating the gut–metabolic axis (71, 72). HEE significantly reduces hepatic lipid accumulation in HFD-induced NAFLD mice, improves liver function, lowers serum inflammatory cytokines and lipid levels, and regulates blood glucose. HEE improves intestinal health by promoting triglyceride hydrolysis, inhibiting fatty acid synthesis, and increasing intestinal short-chain fatty acid production. In addition, HEE increases the abundance of beneficial microbial taxa (*Bacteroidetes* and *Firmicutes*) and enhances overall gut microbiota diversity (*Lachnospiraceae*, *Lactobacillus*, *Ruminiclostridium 9*, and *Romboutsia*) (60). The study has shown that HTF significantly reduce serum TG, TC, and LDL-C levels, while increasing high-density lipoprotein cholesterol in HFD-induced hyperlipidemic mice. These effects are associated with HTF-mediated improvement of gut microbiota dysbiosis, modulation of gut–liver axis interactions, enhancement of bile acid circulation, and attenuation of hepatic lipid accumulation and hyperlipidemia (48). Hawthorn pectin and its POS significantly ameliorate HFD-induced body weight gain, dyslipidemia, and glucose homeostasis imbalance in mice, increase serum glucagon-like peptide-1 levels, and improve insulin sensitivity. Meanwhile, POS helps restore gut microbiota diversity and reduce harmful pathogenic bacteria (such as *Enterococcus*, *Anaerobacterium*, *Lactobacillus* and *Staphylococcus*), and enhance intestinal barrier function, thereby improving overall gut health (73) (Figure 3).

## 5 Safety and translational perspectives of hawthorn in lipid metabolic regulation

As a traditional medicinal and edible plant, hawthorn has a long history of dietary consumption, providing an important foundation for its safety evaluation. However, in the context of modern prevention and management of metabolic diseases, it remains necessary to systematically assess its safety profile and application forms.

### 5.1 Safety evaluation of hawthorn

Hawthorn has been used in China for more than a thousand years, with early documentation dating back to *Xinxiu Bencao* (659 AD) of the Tang dynasty. At present, hawthorn is commonly consumed as fresh fruit, decoction pieces, herbal teas, or components of traditional Chinese medicine formulations for digestive regulation and lipid modulation. From the perspective of traditional medicine, hawthorn is considered safe at conventional dosages, providing a critical prerequisite for its use as a long-term metabolic intervention. Modern scientific study further supported this assessment. In one study, Sprague–Dawley (SD) rats were administered hawthorn extract by oral gavage at doses of 2.25, 4.50, and 9.00 g/kg for 90 consecutive days to evaluate long-term safety. Throughout the experimental period, both male and female animals in all groups exhibited normal growth, with no treatment-related signs of toxicity. Ophthalmic examinations, hematological parameters, serum biochemistry, and routine urinalysis revealed no treatment-related abnormalities. Histopathological evaluation of major organs showed no compound-related lesions, and no subchronic toxic effects were observed at any tested dose. These findings indicate that the no-observed-adverse-effect level of hawthorn extract in SD rats corresponds to approximately 300 times the recommended human intake, suggesting a wide safety margin (74). A separate study investigating the protective effects of *Crataegus azarolus* fruit decoction extract against acetic acid-induced hepatic and renal injury in rats also demonstrated a high safety profile, with a median lethal dose (LD_50_) exceeding 3,500 mg/kg (75). In a real-world study involving 132 people, no adverse reactions were reported after 40 days of consuming hawthorn beverage (500 mL/day, containing 278.7 mg of flavonoids) (76). Collectively, these findings suggest that hawthorn possesses a broad safety window for applications in metabolic regulation research.

### 5.2 Application prospects in functional foods and nutritional interventions

Owing to its favorable safety profile, hawthorn exhibits broad application prospects in the fields of functional foods and nutritional interventions. Such products not only improve palatability but also expand the feasibility of long-term consumption. A study employing the Caco-2 cell monolayer transport model evaluated the lipid absorption–inhibitory activity of a functional beverage formulated with hawthorn proanthocyanidin extract. The results demonstrated that the hawthorn juice beverage significantly reduced oleic acid -induced TG transport across Caco-2 cell monolayers (77). A clinical study investigating a hawthorn fruit extract beverage in Chinese patients with mild hypertension and/or hyperlipidemia showed that after 8 weeks of consumption of a hawthorn beverage containing 19% sucrose, no abnormalities in liver enzymes (alkaline phosphatase and alanine aminotransferase) were observed, and increases in body weight and blood glucose were relatively modest. Although the hawthorn beverage did not demonstrate significant antihypertensive or lipid-lowering effects, it was associated with fewer metabolic adverse reactions than the placebo, with only a small number of participants reporting mild gastrointestinal discomfort (78). The authors suggested that the added sucrose in the beverage may have offset the potential metabolic benefits of hawthorn extract, and hypothesized that calorie-free hawthorn formulations may be more effective in exerting favorable metabolic effects.

In addition, advances in gut microbiota research have promoted the development of microbiota-targeted nutritional intervention strategies. As a natural food capable of modulating gut microbiota composition, hawthorn is expected to play an important role in future “nutrition–microbiota–metabolism” intervention frameworks. A traditional Chinese medicine–probiotic composite preparation (TCMP), consisting of lotus leaf, hawthorn, Kuding tea, and *Bifidobacterium animalis* subsp. *lactis* BPL-1, was shown to significantly reduce HFD-induced body weight gain and lipid accumulation in epididymal, mesenteric, and inguinal adipose tissues in mice, while improving hepatic lipid metabolism, glucose homeostasis, and hepatocellular steatosis. Multi-omics association analyses revealed that TCMP exerts multi-target synergistic effects on HFD-induced metabolic abnormalities by modulating gut–liver axis signaling through the “gut microbiota–short chain fatty acids (SCFAs)–hepatic metabolism” pathway (79).

## 6 Limitations of current studies and future research perspectives

Although studies investigating the role of hawthorn in regulating lipid metabolic homeostasis have increased substantially in recent years and the underlying mechanisms have become progressively clearer, multiple limitations remain when evaluated from the perspectives of evidence-based medicine and systems biology. These issues not only compromise the comparability and reproducibility of existing findings but also, to some extent, hinder the translation of hawthorn-related research outcomes into clinical applications and nutritional interventions. Therefore, a systematic critical appraisal of current studies is warranted, along with the identification of key priorities for future research.

### 6.1 Limitations of research models and experimental design

At present, research on hawthorn-mediated regulation of lipid metabolic homeostasis relies predominantly on *in vitro* experiments and animal models. Although such studies are valuable for elucidating molecular mechanisms, they inherently face limitations when extrapolating findings to human metabolic systems. First, commonly used HFD animal models can replicate certain features of lipid metabolic disorders but fail to fully capture the complexity of human metabolic syndrome, including genetic heterogeneity, lifestyle factors, and long-term disease progression. Second, while *in vitro* cell models offer high controllability for dissecting signaling pathways, they lack critical multi-organ interactions—such as those along the gut–liver axis—making it difficult to recapitulate the multi-target and systemic regulatory effects of hawthorn under physiological conditions. Collectively, these considerations underscore the urgent need for further optimization of model selection and experimental design in future studies.

### 6.2 Insufficient depth and integration of mechanistic studies

Although existing studies have identified the involvement of multiple signaling pathways—such as AMPK, PPAR, SREBP, and FXR—in hawthorn-mediated regulation of lipid metabolic homeostasis, most investigations remain confined to “single-pathway validation” and lack systematic analysis of the interactions among these pathways. Current studies examining the effects of hawthorn on gut microbiota are largely correlational, making it difficult to establish causal relationships between specific microbial alterations and metabolic improvements. Moreover, inconsistencies in reported microbiota alteration patterns across studies may be attributable to differences in host species, dietary composition, and analytical methodologies. Distinguishing between direct effects of hawthorn on host metabolism and indirect effects mediated by the gut microbiota remains a major challenge in this field. Future studies may employ strategies such as antibiotic depletion, fecal microbiota transplantation, and integrated multi-omics analyses to more clearly delineate the causal links among hawthorn, the gut microbiota, and lipid metabolism.

### 6.3 Insufficient clinical evidence and limited levels of evidence

From an application-oriented perspective, the primary bottleneck in translating hawthorn-mediated regulation of lipid metabolic homeostasis lies in the paucity of robust clinical evidence. Most available clinical studies are small-scale and short-term intervention trials, with heterogeneous study designs and outcome measures, making it difficult to generate high-level evidence according to evidence-based medicine standards. In addition, current clinical investigations generally lack mechanism-oriented designs and do not concurrently assess molecular signaling pathways or gut microbiota alterations, thereby limiting direct validation of findings derived from basic research. This limitation consequently weakens the overall persuasiveness and translational impact of existing research findings.

## 7 Conclusion

Hawthorn should not be regarded merely as a “natural lipid-lowering agent,” but rather as a bioactive plant with distinctive value arising from its capacity for multi-target and multi-pathway synergistic regulation in maintaining lipid metabolic homeostasis and ameliorating the pathological features of metabolism-related diseases. Hawthorn, as an auxiliary intervention food material for metabolic diseases, reduces the risk of disease progression through multi-target synergistic regulation. Its favorable safety profile further provides an important guarantee for long-term intervention. The molecular targets and signaling networks underlying the effects of hawthorn and its derived products are becoming increasingly well defined, thereby establishing a solid foundation for the development of functional foods and nutrition-based interventions. Future efforts should focus on overcoming current research limitations and accelerating the translation of hawthorn from basic research into clinical application and industrial development, ultimately providing safer and more effective natural solutions for the prevention and management of lipid metabolism-related diseases.

## Glossary

ACC: acetyl-coenzyme A carboxylase
ACLY: ATP-citrate lyase
ACO: Acyl-CoA oxidase
ADPN: adiponectin
AKT: protein kinase B
ALT: alanine aminotransferase
AMPK: AMP-activated protein kinase
AST: aspartate aminotransferase
ATGL: adipose triglyceride lipase
C/EBP*α*: CCAAT/enhancer-binding protein α
CPT1: carnitine palmitoyltransferase 1
DGAT1: diacylglycerol O-acyltransferase 1
FAS: fatty acid synthase
FXR: farnesoid X receptor
GLUT4: glucose transporter type 4
HFD: high-fat diet
HSL: hormone-sensitive lipase
IL-6: interleukin-6
IRS1: insulin receptor substrate 1
LDL-C: low-density lipoprotein cholesterol
LMD: lipid metabolic disorder
LPS: lipopolysaccharide
NAFLD: non-alcoholic fatty liver disease
Nrf2: nuclear factor erythroid 2-related factor 2
PI3K: phosphoinositide 3-kinase
POS: pectic oligosaccharides
PPAR: peroxisome proliferator-activated receptor
SCD-1: stearoyl-CoA desaturase-1
SCFAs: short chain fatty acids
SREBP-1c: sterol regulatory element-binding protein 1c
TC: total cholesterol
TG: triglyceride
TNF-α: tumor necrosis factor-alpha
VLDL: very-low-density lipoproteins
